# Internet-Delivered Exposure-Based Therapy for Symptom Preoccupation in Atrial Fibrillation: Uncontrolled Pilot Trial

**DOI:** 10.2196/24524

**Published:** 2021-03-02

**Authors:** Josefin Särnholm, Helga Skúladóttir, Christian Rück, Sofia Klavebäck, Eva Ólafsdóttir, Susanne S Pedersen, Frieder Braunschweig, Brjánn Ljótsson

**Affiliations:** 1 Department of Clinical Neuroscience Division of Psychology Karolinska Institutet Stockholm Sweden; 2 Department of Medicine Cardiology Unit Karolinska Institutet Stockholm Sweden; 3 Department of Clinical Neuroscience, Karolinska Institutet Centre for Psychiatry Research Stockholm Health Care Services, Stockholm County Council Stockholm Sweden; 4 Department of Medicine, Cardiology Unit, Karolinska Institutet Department of Cardiology Karolinska University Hospital Stockholm Sweden; 5 Department of Psychology, University of Southern Denmark Department of Cardiology Odense University Hospital Odense Denmark

**Keywords:** atrial fibrillation, arrhythmia, cognitive behavior therapy, quality of life, anxiety

## Abstract

**Background:**

Atrial fibrillation (AF) is the most common cardiac arrhythmia in the adult population. AF is associated with a poor quality of life (QoL) and, in many patients, current medical treatments are inadequate in alleviating AF symptoms (eg, palpitations). Patients often present with symptom preoccupation in terms of symptom fear, avoidance, and control behaviors. Internet-delivered cognitive behavior therapy is effective for treating other somatic disorders but has never been evaluated in patients with AF.

**Objective:**

The aim of this study is to evaluate the efficacy and feasibility of AF-specific internet-delivered cognitive behavior therapy.

**Methods:**

We conducted an uncontrolled pilot study in which 19 patients with symptomatic paroxysmal AF underwent internet-delivered cognitive behavior therapy. Participants completed self-assessments at pretreatment, posttreatment, and at a 6-month follow-up along with handheld electrocardiogram measurements with symptom registration. The treatment lasted 10 weeks and included exposure to physical sensations, reduction in avoidance behavior, and behavioral activation.

**Results:**

We observed large within-group improvements in the primary outcome, AF-specific QoL (Cohen *d*=0.80; *P*<.001), and in symptom preoccupation (Cohen *d*=1.24; *P*<.001) at posttreatment; the results were maintained at the 6-month follow-up. Treatment satisfaction and adherence rates were also high. We observed an increased AF burden, measured by electrocardiogram, at the 6-month follow-up, but a significant decrease was observed in the overestimation of AF symptoms at posttreatment and 6-month follow-up. Exploratory mediation analysis showed that a reduction in symptom preoccupation mediated the effects of internet-delivered cognitive behavior therapy on AF-specific QoL.

**Conclusions:**

This study presents preliminary evidence for the potential efficacy and feasibility of a novel approach in treating patients with symptomatic AF with internet-delivered cognitive behavior therapy.

**Trial Registration:**

ClinicalTrials.gov NCT02694276; https://clinicaltrials.gov/ct2/show/NCT02694276

## Introduction

Atrial fibrillation (AF) is the most common cardiac arrhythmia, with a prevalence of approximately 3% in the general adult population [1]. AF is caused by disturbances in the heart’s electrical signaling, resulting in irregular and often rapid heartbeats, and is associated with a wide range of symptoms such as palpitations, dizziness, fatigue, chest pain, and dyspnea [2]. AF episodes often have a sudden onset and may last briefly or for days; moreover, AF is classified as paroxysmal if the conversion back to the normal sinus rhythm occurs spontaneously within a week [3]. Current treatment strategies (pharmacological and invasive therapies) are associated with potentially serious side effects and do not sufficiently reduce the symptom burden in many patients with AF [4,5]. AF is associated with a poor quality of life (QoL) [6], an increased risk for developing anxiety disorders and depression [7], and high rates of hospitalization and health care utilization, further leading to high societal costs [8]. The QoL impairment in AF has been shown to be unrelated to the objectively measured arrhythmia burden (frequency and duration of AF episodes) [9] and objective measures of disease severity (eg, cardiac dysfunction) [10].

AF burden can be defined as either objective AF episodes, measured and confirmed by an electrocardiogram (ECG), or subjectively perceived AF symptoms measured by self-report [11]. When comparing ECG-recorded episodes of AF and patients’ perceptions of symptoms, patients have been shown to both over- and underestimate the actual burden of AF [11]. Psychological distress has been shown to be predictive of overestimating AF symptoms, that is, indicating AF symptoms while in a normal cardiac rhythm [11-13], a discordance that may be explained by the presence of symptom preoccupation in patients with AF.

We have defined symptom preoccupation in AF as the fear of experiencing and triggering AF episodes, hypervigilance toward cardiac symptoms, persistent worry about complications (eg, stroke), and avoidance of physical and social activities [14]. Symptom preoccupation has shown to be a strong predictor of higher self-reported symptom severity, poor mental and physical QoL, and psychological distress in AF [15] and other somatic and functional disorders [16]. There are several possible pathways through which symptom preoccupation may impact clinical outcomes in AF. Fear and hypervigilance of cardiac-related symptoms may increase the likelihood that patients with AF misinterpret the normal cardiac activity or cardiac activity related to stress as arrhythmia. The perception of AF symptoms may trigger anxiety, leading to autonomic arousal and increases in heart rate, which can trigger extra beats and potential AF episodes [17]. When anticipating or experiencing AF symptoms, behavioral responses—such as avoidance behavior, symptom control behaviors, and excessive worry—may increase the awareness of symptoms [18] and maintain the fearful responses to AF symptoms [19], leading to more AF-related disability. Cardiac anxiety, driven by avoidance behavior, has been shown to affect clinical outcomes by increasing the risk of adverse cardiac prognosis in other heart diseases [20]. In summary, current evidence indicates that psychological and behavioral factors influence clinical outcomes in AF and that AF disability extends beyond the actual arrhythmia.

Cognitive behavior therapy (CBT), with an emphasis on exposure, aims to break the cycle of avoidance behavior, symptom fear, and disability through systematic contact with a stimulus that evokes conditioned aversive responses while abstaining from avoidance and safety behaviors [21]. Exposure therapy also encourages patients to willingly expose themselves to aversive stimuli by simultaneously engaging in a behavior that is inconsistent with the emotional response elicited by that stimuli [22]. Our research group has developed and evaluated an AF-specific CBT protocol. In a previous study, we conducted an investigation of exposure-based AF-specific CBT in a face-to-face format targeting symptom preoccupation. It showed promising effects on AF-specific QoL and self-reported AF symptoms [14].

One development in the effort to bridge the gap between the demand and availability of CBT [23] has been the delivery of treatment via the internet. Internet-CBT has been evaluated in a range of trials for somatic and psychiatric disorders, with large treatment effects [24], but has never been evaluated for AF. Studies on internet-CBT in patients with cardiac disorders are scarce [25], and there has been a call to increase access to digital health solutions in the cardiac health care community [26].

The purpose of this study is to evaluate the efficacy and feasibility of internet-delivered AF-specific CBT, primarily based on exposure exercises in preparation for a forthcoming randomized controlled trial (RCT). Additional objectives of the study are to investigate changes over time in the objectively measured AF burden and in the participants’ potential overestimation of their AF symptoms as well as to explore whether symptom preoccupation was a potential mediator of treatment effect on AF-specific QoL.

## Methods

### Design Overview

This is an uncontrolled pilot study with a pretest-posttest design and a 6-month follow-up. We aimed to recruit 30 participants to achieve a power of 80% for detecting a moderate improvement, as indicated by an effect size of Cohen *d*=0.65, in the primary outcome measure of disease-specific QoL between pre- and posttreatment assessments. However, only 19 participants were recruited because of a slow recruitment rate. An interim analysis after 14 participants had been recruited showed significant improvements on the main outcome measure; therefore, we judged the discontinuation of recruitment to be justifiable.

### Participants

The participants were referred by cardiologists in Stockholm, Sweden. To be eligible for this study, participants had to have (1) paroxysmal AF as defined above and (2) receive optimal medical care according to the current clinical guidelines [3]. The following inclusion criteria also had to be fulfilled: (1) ≥1 AF episode per month and symptoms that troubled the patient or caused limitations in daily activities (ie, European Heart Rhythm Association [EHRA] class ≥IIb; [27]); (2) age 18-75 years; and (3) ability to read and write in Swedish. The exclusion criteria were as follows: (1) heart failure with severe systolic dysfunction (ejection fraction ≤35%); (2) significant valvular disease; (3) planned ablation for AF or ablation during the 3 months before assessment; (4) other severe medical illness; (5) any medical restriction on physical exercise; (6) severe psychiatric disorder, severe depression, or risk of suicide; and (7) alcohol dependency. Participants were asked not to participate in any concurrent psychological treatment during the course of this treatment and were encouraged, along with their referring cardiologists, to avoid changes in medical therapy unless clinically necessary. The study was approved by the regional ethical review board in Stockholm, Sweden (reg.no: 2015/1843-31/2) and was registered on ClinicalTrials.gov (NCT02694276). This report of the study adheres to the TREND Statement checklist for nonrandomized interventions [28].

### Instruments

The primary outcome was the Atrial Fibrillation Effect on Quality-of-Life (AFEQT), which is an AF-specific measure of QoL that evaluates the following parameters: AF symptoms, impairment in social and physical activities, medical treatment concerns, and AF-specific treatment satisfaction [29]. The scale has 20 items, with the total score ranging from 0 (severe symptoms and disability) to 100 (no symptoms and disability). The total AFEQT score corresponded to the following categories of AF severity: mild (71.3, SD 20.6), moderate (57.9, SD 19.0), and severe (42.0, SD 21.2) [29]. The subscale *AF-specific treatment satisfaction* (two items) measures satisfaction with the current medical treatment and is not included in the total score; thus, it was not analyzed in this study.

Secondary outcome measures included the Symptoms Checklist, Frequency, and Severity Scale (SCL), which is a disease-specific checklist used to measure AF-related symptoms on two subscales - symptom severity and symptom frequency [30]. The University of Toronto Atrial Fibrillation Severity Scale was used to assess AF-specific symptoms [31] and AF-specific health care utilization. From that scale, we combined items 10 (visits to emergency room), 11 (number of hospitalizations), and 12 (visits to cardiologists) to assess cardiac-specific health care consumption. The Cardiac Anxiety Questionnaire (CAQ) measures cardiac anxiety using 3 subscales: (1) fear (eg, When my heart is beating fast, I get frightened), (2) avoidance (eg, I avoid exercise or other physical work), and (3) attention (eg, I pay attention to my heartbeat) [32] with a greater score indicating an elevated cardiac anxiety.

The 4-item Perceived Stress Scale (PSS-4) was used to assess stress sensitivity, [33], the 7-item Generalized Anxiety Disorder (GAD-7) scale was used to measure general worry and anxiety [34], and the Patient Health Questionnaire (PHQ-9) was used to measure depressive symptoms [35]. Participants also completed the World Health Organization Disability Assessment Schedule (WHODAS), a well-validated measure of general health and disability [36].

The Client Satisfaction Questionnaire (CSQ-8) was used to measure satisfaction with the CBT treatment [37]. At posttreatment and 6-month follow-up, we assessed whether participants had experienced any adverse events caused by their participation in the treatment. Participants were asked to report and rate the short- and long-term discomfort of the adverse event on a scale of 0 (*did not affect me at all*) to 3 (*affected me very negatively*) [38]. Other measures were also included, but these are not presented in this paper.

### ECG Measurements

To assess the objective AF burden, participants were asked to perform a 30-second intermittent handheld ECG (Zenicor EKG thumb, Stockholm, Sweden) registration at home, four times daily, and while experiencing cardiac symptoms over 2 weeks at pre-and posttreatment and at 6-month follow-up. The participants were instructed to push a symptom indicator button on the device when they perceived that they had AF symptoms, both during the regular four daily measurements and while experiencing cardiac symptoms. A more detailed technical description of the device is available elsewhere [39].

### Intervention

The AF-specific exposure-based internet-CBT treatment was delivered completely via the internet through a tailored and secure web-based platform. The treatment was therapist-guided, lasted for 10 weeks, and included 5 interactive treatment modules with weekly homework assignments to be completed during the first 5 weeks. The modules were downloadable as PDF files and comprised between 13 and 16 pages (A4) of text, and a total of 68 pages. After the fifth module, participants continued to work with the treatment content for the remaining 5 weeks and sent weekly reports about their exposure exercises to their treating psychologist. Participants were encouraged to work with the treatment for approximately 30-60 minutes per day.

The treatment protocol was based on an AF-specific CBT protocol that was previously evaluated in a face-to-face pilot study [14] and an exposure-based CBT manual for irritable bowel syndrome [38]. The treatment primarily targets two maintenance processes of AF disability: hypervigilance and fear of AF symptoms and the avoidance of physical and social activities. The treatment comprised the following interventions: (1) education on AF (pathophysiology and medical treatment) and symptom preoccupation; (2) a self-observation exercise (ie, awareness of current cardiac symptoms, thoughts, feelings, and behavioral impulses to reduce the negative valence of symptoms); (3) exposure to physical sensations similar to AF symptoms by engaging in a variety of physical exercises, such as increasing the heart rate by running up and down the stairs or inducing dizziness or dyspnea by overbreathing to reduce symptom-related fear and hypervigilance; (4) in vivo exposure to avoided situations or activities where symptoms are unwanted (such as participating in a social activity while experiencing cardiac symptoms); (5) reduction or removal of behaviors, such as postponing pulse checking, that serve to control symptoms. Interventions (2)-(6) were used in conjunction to maximize their effectiveness, for example, participants were encouraged to use the self-observation exercise during exposure and to enhance exposure by inducing physical sensations while conducting exposure in vivo. AF episodes that occurred during treatment were also framed as an opportunity to practice the new skills acquired via the treatment. Examples of AF-related avoidance and control behaviors and how these could be targeted with exposure exercises were also presented in comprehensive clinical vignettes; (7) behavioral activation, where patients were encouraged to take steps toward goals within important life areas impaired by AF; and (8) in the final module, participants worked with relapse prevention to handle the potential progression of AF. The treatment is described in more detail in the report of our previous pilot study [14]. Textbox 1 provides an overview of the treatment components.

**Overview of treatment components.**

Education

- Pathophysiology of atrial fibrillation (AF).
- The role of anxiety and behavior on symptoms and quality of life.
- Brief training in self-observation.

Interoceptive exposure

- Exposure to physical sensations similar to AF symptoms.

Exposure in vivo

- Gradual exposure to avoided situations or activities that patients fear may trigger AF symptoms or where symptoms are unwanted.
- Combining in vivo exposure with interoceptive exposure.

Behavioral activation

- Identifying life areas impaired by AF-related disability or symptom fear.
- Set behavioral goals and gradually take steps toward them.

Relapse prevention

- Prevention of relapse into control or avoidance behaviors by identifying risk situations.

### Therapist Support

The treatment was delivered by two clinical psychologists (BL and JS) with thorough training in exposure-based CBT and experience in treating AF. The participants had continuous contact with an assigned therapist throughout the 10-week treatment period. The role of the therapist was to guide the patient through the treatment and provide feedback on the homework assignments. To progress to the next treatment module, the participants had to complete their weekly home assignments (eg, read the treatment module and conduct the exposure exercises) and report it to the therapist. In addition to reporting the weekly homework assignments, participants could send questions to their therapist via an asynchronous messaging system. Participants received feedback on their reports and questions within two working days. If a participant had technical problems with the platform or did not respond for more than a week, a phone call was made. No treatment interventions were administered over the phone. The treating psychologists could also consult the study cardiologists (FB and HS) at any point throughout the treatment if there were questions regarding the participants’ physical health.

### Procedure

Cardiologists within tertiary care were informed about the study via email and lectures and referred participants to the study. The referring cardiologists signed a health form where they confirmed fulfillment of the inclusion criteria, meaning that they confirmed the AF diagnosis and the classification of EHRA class ≥IIb; [27]; certified that the participant had undergone a thorough cardiac evaluation including a recent echocardiography (<12 months old; ejection fraction >35%); and ensured that the participants’ medical treatment was in accordance with the current guidelines [3] and that the participants had no contraindications to being physically active. Eligible patients underwent a telephone-based structured psychological assessment by a clinical psychologist (JS) and completed the Alcohol Use Disorders Identification Test [40] and PHQ-9 [35]. Participants then underwent a telephone-based clinical interview by a final-year medical student (SK) who also screened the participants’ medical charts. In case of uncertainty regarding the participants’ physical health, the study cardiologists (FB, HS) were consulted before a decision on inclusion was made, and informed consent was obtained. All self-rated measures were completed over the internet using a secure assessment tool, and the outcomes were collected at pretreatment, posttreatment, and 6-month follow-up. In addition, a shortened version of AFEQT (AFEQT-S; Multimedia Appendix 1), CAQ, and PSS-4 were collected weekly during treatment.

### Statistical Analysis

All analyses were performed using R [41]. Pretreatment, posttreatment, and 6-month follow-up data were included in a piecewise linear mixed-model analysis with a random intercept. Separate group-level slopes were estimated for the pre- to posttreatment assessment (Slope 1) and posttreatment to the 6-month follow-up assessment (Slope 2). A significant Slope 1 was interpreted as a treatment effect. A nonsignificant Slope 2 was interpreted as the maintenance of improvement during the follow-up period, whereas a significant Slope 2 was interpreted as improvement or deterioration during the follow-up period. Slope 1 and Slope 2 were then summed to form the estimated overall pre- to 6-month follow-up improvement. Effect sizes (Cohen *d*) were calculated by dividing the estimated pairwise differences between the three time points by the model-implied standard deviation at pretreatment. Effect sizes were categorized according to Cohen recommendations, with small, medium, and large effect sizes corresponding to *d*=0.20, 0.50, and 0.80, respectively [42]. To account for the nonnormal distribution of d [43], 95% CIs for the effect sizes were obtained from 5000 bootstrap replications of the mixed-model analyses.

The ECG measurements were used to analyze the change in AF burden from pretreatment to posttreatment and 6-month follow-up as well as to investigate changes in AF symptom overestimation (ie, indicating AF symptoms on the device when in sinus rhythm). Multimedia Appendix 1 shows a further description of the ECG analysis.

Finally, we performed exploratory mediation analyses to investigate whether changes in symptom preoccupation were a potential mediator of the treatment effect on QoL. We included the three subscales of CAQ (attention, avoidance, and fear) as the indicators of symptom preoccupation, and we also included PSS-4 as a competing mediator. PSS-4 measures stress sensitivity, which was not targeted by the CBT intervention, and by including it that as a competing mediator, we could control for nonspecific improvements. Multimedia Appendix 1 shows for a further description of the mediation analysis.

## Results

### Sample

**Figure 1:**
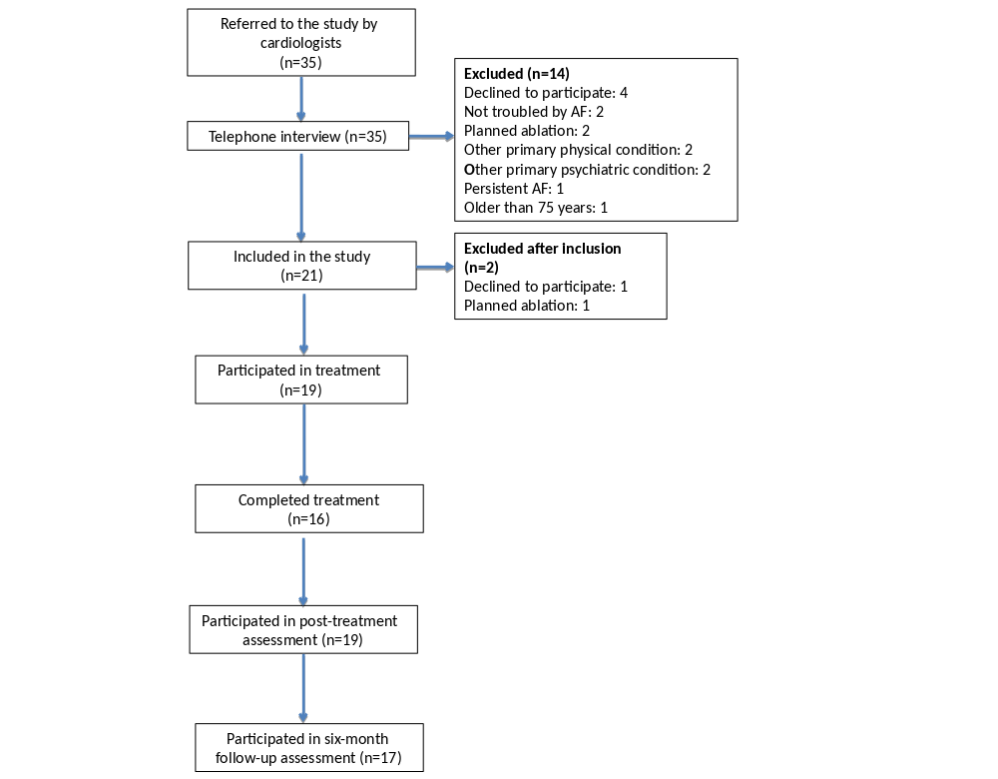
Patient flow through the study. AF: atrial fibrillation.

The study comprised 19 participants (12 women and seven men), who were recruited and treated between January 2016 and January 2018. Figure 1 shows the patient flow through the study. The mean age was 60.9 (SD 9.6) years, and the reported mean time since the diagnosis of AF was 6.2 (SD 8.4) years. Table 1 shows the demographics of participants.

During the analysis of the ECG data that were obtained after inclusion, we discovered that two participants had persistent AF at the pretreatment assessment, which means that they, in retrospect, did not fulfill the inclusion criteria of paroxysmal AF at the pretreatment assessment; however, both the participants and their treating physician had reported paroxysmal AF during the inclusion process and were also assessed by the research team as such. Therefore, we decided to keep these two participants in the analysis.

**Table 1:** Patient demographics.

Characteristics		Values
Women, n (%)		12 (63)
Age (in years), mean (SD)		60.9 (9.6)
**Employment status, n (%)**		
	Employed	7 (37)
	Retired	8 (42)
	Unemployed	1 (5)
	Sick leave	3 (16)
	In partnership	9 (47)
**Highest completed education, n (%)**		
	Vocational	3 (16)
	Post-secondary	3 (16)
	Tertiary	13 (68)
AF^a^ duration, mean (SD)		6.2 (8.4)
Previous ablation, n (%)		5 (25)
Pacemaker, n (%)		2 (11)
**Current medication, n (%)**		
	Antiarrhythmics	7 (37)
	Beta blockers	16 (84)
	Anticoagulation	13 (68)
	ACEi^b^/ARB^c^	5 (26)
	Anxiolytics	3 (16)
	Sleep medication	4 (21)
**Medical disorders, n (%)**		
	Previous stroke/TIA^d^	3 (16)
	Diabetes	2 (11)
	Obstructive sleep apnea	3 (16)
	Hypertension	5 (26)
	Hypothyroidism	1 (5)
	Any medical disease	13 (68)
	Previous psychological treatment	12 (63)
	Current psychiatric disorder	4 (21)

^a^AF: atrial fibrillation.

^b^ACEi: angiotensin-converting enzyme inhibitor.

^c^ARB: angiotensin receptor blocker.

^d^TIA: transient ischemic attack.

### Treatment Activity

In total, 16 out of 19 participants (84%) were considered as treatment completers after completing at least four modules of the treatment and thus commenced work with exposure exercises. The noncompleters (n=3) completed 2-3 modules. The mean number of messages that participants sent and received from their treating psychologist were 12.0 (SD 7.1; range 1-25) and 15.7 (SD 6.0; range 1-27), respectively. The psychologist spent a weekly mean of 9.5 minutes (SD 6.4; range: 4-23 min) per patient.

### Primary and Secondary Outcomes

Table 2 displays scores on the continuous outcome measures at the assessment points together with *P* values of slope estimates and effect sizes (d) with 95% bootstrap CIs. Participants showed significant improvements in AF-specific QoL, as measured by AFEQT, with large within-group effect sizes posttreatment (d=0.80) and at 6-month follow-up (d=0.72) as compared with baseline, whereas there was no significant difference between posttreatment and 6-month follow-up. The total AFEQT score corresponded to moderate AF severity at baseline; however, at posttreatment and follow-up, the total AFEQT score corresponded to mild AF severity. We observed large effect sizes posttreatment (d=1.43) and at 6-month follow-up (d=1.52) in the reduction of cardiac anxiety (CAQ). All three subscales of CAQ (attention, avoidance, and fear) showed a significant improvement posttreatment and at 6-month follow-up. We observed moderately sized pre- to posttreatment improvements in depression (PHQ-9), general anxiety (GAD-7), perceived stress (PSS-4), and general QoL (WHODAS). Furthermore, we observed a medium-sized improvement in self-reported AF symptom severity and a small improvement in AF symptom frequency (SCL scales) and general AF symptoms (AFFS) from pre- to posttreatment. We observed a medium effect size in the reduction of cardiac-specific health care visits posttreatment and a further reduction with a large effect size at 6-month follow-up.

**Table 2:** Continuous treatment outcome measures.^a^

Outcome measure	Observed outcomes				Contrasts							
	Pretreatment, mean (SD)	Posttreatment, mean (SD)	Follow-up, mean (SD)	Pre to post				Pre to follow-up			Post to follow-up	
				*P* value		Cohen *d* (95% CI)	*P* value		Cohen *d* (95% CI)	*P* value		Cohen *d* (95% CI)
AFEQT^b^	66.8 (18.9)	81.0^c^ (17.4)	82.6^c^ (18.3)	<.001		0.80 (0.45 to 1.24)	<.001		0.72 (0.32 to 1.23)	.56		−0.08 (−0.28 to −0.12)
CAQ^d^ total	32.5 (10.2)	19.5^c^ (9.2)	18.6^c^ (8.0)	<.001		1.43 (0.91 to 1.94)	<.001		1.52 (1.07 to 2.05)	.67		0.10 (−0.17 to 0.45)
CAQ fear	16.1 (5.8)	9.4^c^ (4.1)	9.3^c^ (3.7)	<.001		1.45 (0.88 to 2.00)	<.001		1.49 (0.93 to 2.10)	.79		0.07 (−0.27 to 0.42)
CAQ avoidance	6.3 (4.3)	3.8^c^ (3.8)	3.1^e^ (2.8)	.003		0.68 (0.21 to 1.25)	.001		0.75 (0.29 to 1.27)	.72		0.08 (−0.15 to 0.33)
CAQ attention	10.1 (3.7)	6.3^c^ (3.1)	6.2^c^ (3.4)	<.001		1.12 (0.64 to 1.67)	<.001		1.20 (0.71 to 1.73)	.72		0.08 (−0.18 to 0.40)
SCL^f^ frequency	19.2 (10.9)	15.8^g^ (9.9)	15.2^g^ (10.2)	.013		0.33 (0.09 to 0.78)	.01		0.34 (0.07 to 0.70)	.91		0.01 (−0.25 to 0.20)
SCL severity	21.2 (6.3)	19.0^f^ (3.6)	19.2^f^ (4.2)	.02		0.46 (0.12 to 0.88)	.03		0.41 (0.04 to 0.94)	.81		−0.04 (−0.32 to 0.18)
AFSS^h^	10.3 (7.8)	8.1 (6.6)	7.6 (6.5)	.08		0.27 (0.02 to 0.72)	.09		0.27 (−0.06 to 0.66)	.998		0.00 (−0.32 to 0.18)
AFSS health care visits	1.3 (2.0)	0.6^g^ (0.8)	0.2^e^ (0.4)	.046		0.56 (0.12 to 0.92)	.006		0.82 (0.33 to 1.25)	0.36		0.26 (−0.08 to 0.48)
PHQ-9^i^	6.1 (5.4)	3.1^e^ (3.3)	3.9^g^ (2.8)	.002		0.75 (0.35 to 1.32)	.02		0.57 (0.13 to 0.91)	.42		−0.18 (−0.52 to 0.11)
GAD-7^j^	6.1 (5.8)	3.4^e^ (3.1)	3.8 (3.3)	.005		0.64 (0.23 to 1.06)	.01		0.58 (0.27 to 0.88)	.78		−0.06 (−0.35 to 0.27)
PSS-4^k^	5.7 (3.4)	4.1^c^ (2.4)	4.6 (2.2)	.006		0.62 (0.23 to 1.07)	.094		0.38 (−0.14 to 0.92)	.28		−0.24 (−0.67 to 0.04)
WHODAS^l^	19.0 (17.0)	12.8^e^ (16.8)	11.0^e^ (14.6)	.004		0.38 (0.12 to 0.86)	.001		0.46 (0.18 to 0.93)	.55		0.08 (−0.21 to 0.22)

^a^Within-group effect sizes (ES; Cohen *d*) and *P*-values are presented for differences between the three assessment points: pretreatment, posttreatment, and 6-month follow-up based on the piecewise linear mixed-model analysis. ES were reported with 95% CIs based on 5000 bootstrap replications.

^b^AFEQT: Atrial Fibrillation Effect on Quality-of-Life.

^c^*P*<.001.

^d^CAQ: Cardiac Anxiety Questionnaire.

^e^*P*<.01.

^f^SCL: Symptoms Checklist, Frequency and Severity Scale.

^g^*P*<.05.

^h^AFSS: University of Toronto Atrial Fibrillation Severity Scale.

^i^PHQ-9: Patient Health Questionnaire-9.

^j^GAD-7: Generalized Anxiety Disorder-7.

^k^PSS-4: Percieved Stress Scale-4.

^l^WHODAS: World Health Organization Disability Assessment Schedule.

### ECG Evaluation

A total of 2243 ECG recordings were documented. Of these, 2189 (97.6%) could be classified as sinus rhythm or AF, 36 (1.6%) could not be classified, and 18 (0.8%) were considered artifacts. One participant contributed only pretreatment observations. The AF burden increased from pretreatment to the 6-month follow-up (OR=1.235; *P*=.02). In the AF overestimation analysis, 2175 observations, of which 143 (6.6%) were symptom indications, were included. We excluded 14 recordings where patients were in sinus rhythm but had an irregular pulse due to premature ventricular contractions, which may cause symptoms similar to those of AF, from the analysis. We observed statistically significant decreases in overestimation proportions at both posttreatment (OR=−1.153, *P*<.001) and 6-month follow-up (OR=−1.538; *P*<.001), compared with the pretreatment assessment. Multimedia Appendix 1 shows the observed AF burden and overestimation of AF symptoms at the three assessment points and odds ratios from the multilevel logistic regressions.

### Mediation Analysis

Mediators and outcomes were collected for weekly measurements for 9 consecutive weeks during treatment. The mean number of observations per week was 16.9 (out of possible 19), and the lowest number of observations was 15 during the eighth week. In the single mediator analysis, all the tested mediators had statistically significant ab-products, thereby indicating that they all explained some part of the improvement during treatment on the AFEQT-S. In the multiple mediator analysis, where the mediators competed with each other in explaining the change in AFEQT-S, the CAQ fear subscale and PSS-4 ab-products were substantially lower than that in the single mediator analysis, and the former was no longer significant. The ab-products for the CAQ attention and avoidance subscales were of equal size, statistically significant, and more than three times larger than the other two mediators in the multiple mediator model. Multimedia Appendix 1 shows the estimated indirect effects, ab-products, and their confidence intervals for the four mediators, when tested separately and together in a multiple mediator model.

### Treatment Satisfaction

At posttreatment, participants scored an average of 24.9 (SD 4.9) out of 32 points on treatment satisfaction as measured by the CSQ-8, and 18 out of 19 participants reported that they were satisfied with the treatment and that it had helped them to deal more effectively with their AF symptoms.

#### Changes in Medication and in Cardiac Health

At follow-up, 2 of 17 participants had made minor changes in their cardiac medication. None of the patients had undergone invasive cardiac procedures between posttreatment and follow-up. At follow-up, one participant reported deterioration in cardiac health due to more frequent AF episodes. The ECG recording at follow-up indicated a progression to persistent AF in one additional patient.

#### Adverse Events

At posttreatment, four participants reported an adverse event from participating in the study. The adverse events described were stress because of the study (n=3) and an increased cardiac attention (n=1). Three of the adverse events were given a low severity rating, both when the effect occurred and on residual discomfort, corresponding to a rating of 1 on a scale of 0-3. One event was rated as having the highest severity (3/3) at the time of occurrence and a (2/3) moderate severity rating for the residual comfort. At the six-month follow-up, no current or residual adverse events from participating in the study were reported.

## Discussion

### Principal Findings

To the best of our knowledge, this is the first study to investigate the efficacy and feasibility of internet-delivered, exposure-based CBT for symptom preoccupation in patients with symptomatic paroxysmal AF. We observed a large improvement in the primary outcome measure of AFEQT that measured AF-specific QoL and self-reported AF symptoms and a large reduction in symptom fear, avoidance behaviors, and hypervigilance as measured by the CAQ. These effects remained large-6 months after the treatment. We also observed small to medium significant effects on the other outcome measures at posttreatment, which were sustained or improved at the 6-month follow-up. Furthermore, the participants’ high adherence to and satisfaction with the treatment, limited reports of adverse events, and perception of being able to deal more effectively with their AF symptoms indicate that exposure-based internet-CBT is a feasible treatment option for the target population.

The results suggest that the AF-specific exposure-based internet-CBT had effects in patients with paroxysmal AF that are comparable with the effects of exposure-based internet-CBT in other somatic functional disorders [38,44-47]. Our results also converge with the results of face-to-face and internet-CBT for patients with cardiovascular disease with anxiety and depression reported in a recent meta-analysis [25] and two recent RCTs [48,49].

We observed a significant increase in the objectively measured AF burden at the 6-month follow-up, which is not unexpected because AF is known to be a progressive disease [50]. Interestingly, the overestimation proportion (ie, when the participant indicated AF symptoms while in normal cardiac rhythm) was significantly decreased both at posttreatment and at 6-month follow-up. Psychological distress has been shown to be predictive of overestimating the AF symptoms [11-13], and it is possible that reduction in symptom fear and hypervigilance improved the patients’ ability to accurately estimate their cardiac rhythm. Despite the observed increase in objective AF burden over time, the AF-specific QoL and symptom preoccupation still showed large improvements and were maintained at the 6-month follow-up. Together with the significant reduction in health care visits, this result indicates that despite the natural progression in AF symptoms, participants did not relapse in symptom preoccupation and were still able to maintain a good QoL.

The mediation analyses showed that a reduction in symptom preoccupation mediated the effect of exposure-based internet-CBT on AF-specific QoL and self-reported AF symptoms. These results are in line with the previous mediation analyses by our group of exposure-based CBT, where we have found that a reduction in avoidance behavior mediates improvement in other chronic health conditions [51-53].

### Limitations

The results of this study should be interpreted with the following limitations kept in mind. Importantly, we did not use a control group, which limits the causal inference that can be drawn from the results. Without a control group, we cannot control for the passage of time, the effect of attention from a caregiver, and expectancy of improvement. More than half of the participants had a previous experience of psychological treatment; thus, it is possible that psychological treatment was more suitable for the referred participants than the average patient with AF. The referral by clinical cardiologists could have contributed to a positive expectancy effect dependent on the caregivers’ attitude toward CBT. It is also possible that patients who were generally less prone to respond to treatment were referred to the study, hence contributing to a selection bias. The sample also had disproportionately more women and the subjects were younger than the general AF population [50], further limiting the generalizability of the results. Another limitation is that the handheld ECG is not a continuous measurement of the cardiac rhythm, which may contribute to undetected AF symptoms remaining undetected.

### Conclusions

This is the first study in which patients with symptomatic paroxysmal AF were treated with therapist-guided exposure-based internet-CBT. Despite already receiving optimal medical therapy at baseline, we observed large improvements in AF-specific QoL and symptom preoccupation that were sustained at the 6-month follow-up. Our study highlights avoidance behavior and hypervigilance as potentially important psychological factors contributing to symptom severity and low QoL in patients with AF. However, the psychological aspects of AF appear to be underrecognized in the current medical literature and clinical practice.

We conclude that symptom preoccupation is an important target for treatment and that the treatment may be both feasible and clinically effective for the target population. AF-specific CBT delivered via the internet has the potential to reduce health care utilization and improve the well-being of a large group of patients, when current treatment methods have limited effectiveness. These results need to be confirmed in RCTs.

## Appendix

Multimedia Appendix 1 Description of electrocardiogram and mediation analysis.

## Abbreviations

AF: atrial fibrillation
AFEQT: Atrial Fibrillation Effect on Quality-of-Life
CAQ: Cardiac Anxiety Questionnaire
CBT: cognitive behavior therapy
CSQ-8: Client Satisfaction Questionnaire
ECG: electrocardiogram
EHRA: European Heart Rhythm Association
GAD-7: Generalized Anxiety Disorder
PHQ-9: Patient Health Questionnaire
PSS-4: Perceived Stress Scale
QoL: quality of life
RCT: randomized controlled trial
SCL: Symptoms Checklist, Frequency and Severity Scale
WHODAS: World Health Organization Disability Assessment Schedule
