# Supplementary material for: Internet-Delivered Exposure-Based Therapy for Symptom Preoccupation in Atrial Fibrillation: Uncontrolled Pilot Trial
Source: JMIR Cardio. 2021 Mar 2;5(1):e24524. doi: 10.2196/24524 (PMC8411432; doi:10.2196/24524)
Supplement: Multimedia Appendix 1 [file cardio_v5i1e24524_app1.docx]

Supplement 1.

**ECG analyses**

To assess the AF symptom burden in the sample at the three assessment points the individual symptom burden was calculated as the number of AF observations divided by the total number of scheduled ECG measurements (4 times per day). The mean, standard deviation, median and interquartile range (IQR) of individual burdens are reported. Analysis of change in symptom from pre-treatment to post-treatment and six-month follow-up was based on multilevel mixed effects logistic regression. Each observation (level 1) was classified as 0 (sinus rhythm) or 1 (atrial fibrillation) and the observations were nested within day (level 2) and individual (level 3). Assessment point was entered as a factor with pre-treatment as the reference level. In the second set of analyses, we investigated AF symptom overestimation. These analyses were based on all ECG observations, including both regular measurements and when patients experienced symptoms. Symptom overestimation was defined as indicating that symptoms were present although the ECG device measured sinus rhythm, which was coded as 1. If the participant did not indicate or correctly indicated AF symptoms, the observation was coded as 0. Individual proportions of overestimation were calculated and the mean, standard deviation, median and IQR of these proportions are reported. Analysis of change in overestimation between the assessment points was also based on multilevel mixed effects logistic regression, but the day level was omitted because including it did not improve model fit.

**Mediation analyses**

The mediation analyses were based on the 9 weekly measurements during the treatment phase, i.e., after the first treatment week until the tenth treatment week. In the mediation analyses, a weekly measured short version of the AFEQT (AFEQT-S) developed by the research group was included as the outcome variable. The recall period of the AFEQT-S was changed to one week instead of one month and the last six questions concerning worry about side effects of and satisfaction with the current medical treatment were removed, as these were not assumed to be targeted by the CBT intervention. Also, question number 6 that asked about the participant’s ability to (1) be in a relationship and (2) participate in activities with friends and family was separated into two questions, because they were considered to measure two distinct types of AF-related disability. The average score of these two questions was used in the rescaled 0-100 summary score for consistency with the full scale. The proposed mediators were the three subscales of CAQ (attention, avoidance, and fear) as indicators of symptom preoccupation and we also included PSS-4 as a competing mediator.

The analyses followed the process outlined by Baron and Kenny (1) and further developed by Preacher and Hayes (2). Two sets of mediation analyses were conducted. In the first set, each of the four mediators were tested separately. In the second set, all mediators were included in the multiple mediator analyses (2) to investigate their relative contribution to improvement in QoL as measured by the AFEQT-S. The premise of the mediation analyses was that there would be an effect of treatment week on the mediators and the outcome (i.e., participants would improve gradually and linearly during the treatment period) and there would be an association between the mediators and the outcome throughout the treatment period. The purpose of the mediation analyses was to estimate how much of the per-week improvement on the outcome was explained by change in the mediators. Each set of analysis was performed in three steps. First, the relationship between treatment week and the mediator(s) (i.e., *a*-path) was estimated. Second, the relationship between the mediator(s) and AFEQT-S (i.e., *b*-path) throughout the treatment period was estimated, controlling for treatment week. Third, the *a* and *b*-path estimates for each mediator were multiplied to form the *ab*-product, which is the indirect, or mediated effect (i.e., how much of the effect of treatment week on the outcome that is explained by change in the mediator). In the second set of analyses, which included all mediators, the first step was conducted separately for each mediator as the dependent variable and the second step included all mediators as independent variables. All analyses were based on linear mixed models with random intercept to account for dependency between the weekly measurements. CIs for the indirect effects, the *ab-*products, were estimated using 5000 bootstrap replications of all analyses and the criterion for statistically significant mediation was that the CI did not contain zero (2).

Table S1. ECG measurements: AF burden and overestimation

| Assessment point | Mean | SD | Median | IQR | OR | *p* |
| --- | --- | --- | --- | --- | --- | --- |
| *AF burden* |  |  |  |  |  |  |
| Pre-treatment | .183 | .348 | .011 | [0, .170] |  |  |
| Post-treatment | .162 | .327 | 0 | [0, .111] | -0.313 | .569 |
| 6-month follow-up | .215 | .382 | .023 | [0, .171] | 1.235 | .022 |
| *AF overestimation* |  |  |  |  |  |  |
| Pre-treatment | .088 | .180 | .019 | [0, .057] |  |  |
| Post-treatment | .045 | .103 | 0 | [0, .028] | -1.153 | <.001 |
| 6-month follow-up | .033 | .106 | 0 | [0, 0] | -1.538 | <.001 |

Note. The table shows the mean and median proportions of AF burden and AF overestimation in the sample at the three assessment points. The OR is the estimated change from pre-treatment according to multilevel mixed effects logistic regression and p-value is the associated statistical significance of the change. Abbreviations: AF = Atrial fibrillation, IQR = Interquartile range, OR = Odds ratio, SD = Standard deviation.

Table S2. Indirect effects, *ab*-product, of the four tested mediators of the effect of treatment week on the primary outcome measure AFEQT-S.

|  | Single mediator  analysis | |  | Multiple mediator  analysis | |
| --- | --- | --- | --- | --- | --- |
| Mediator | *ab* | CI |  | *ab* | CI |
| CAQ attention | 1.22* | [0.77, 2.00] |  | 0.73* | [0.31, 1.35] |
| CAQ avoidance | 0.99* | [0.43, 1.91] |  | 0.73* | [0.33, 1.39] |
| CAQ fear | 0.90* | [0.41, 1.63] |  | 0.14 | [-0.40, 0.64] |
| PSS-4 | 0.51* | [0.24, 1.06] |  | 0.20* | [0.04, 0.57] |

*Statistical significance of indirect effects, *ab*-products, based on their respective CIs not containing zero. Abbreviations: AFEQT-S = Atrial Fibrillation Effect on Quality-of-Life – Short version. CAQ = Cardiac Anxiety Questionnaire, PSS-4 = Perceived stress scale - 4 item version.

References

1. Baron R, Kenny D. The moderator-mediator variable distinction in social psychological research: conceptual, strategic, and statistical considerations. J Pers Soc Psychol. 1986 Dec 1;51(6):1173–82. PMID:3806354

2. Preacher KJ, Hayes AF. Asymptotic and resampling strategies for assessing and comparing indirect effects in multiple mediator models. Behav Res Methods. 2008 Aug;40(3):879–91. DOI:10.3758/BRM.40.3.879
